# A Systematic Review of the Impact of the First Year of COVID-19 on Obesity Risk Factors: A Pandemic Fueling a Pandemic?

**DOI:** 10.1093/cdn/nzac011

**Published:** 2022-04-08

**Authors:** Natasha Faye Daniels, Charlotte Burrin, Tianming Chan, Francesco Fusco

**Affiliations:** School of Clinical Medicine, University of Cambridge, Cambridge, United Kingdom; School of Clinical Medicine, University of Cambridge, Cambridge, United Kingdom; School of Clinical Medicine, University of Cambridge, Cambridge, United Kingdom; Department of Public Health and Primary Care, University of Cambridge, Cambridge, United Kingdom

**Keywords:** COVID-19, obesity, depression, physical activity, financial stress, diet

## Abstract

Obesity is increasingly prevalent worldwide. Associated risk factors, including depression, socioeconomic stress, poor diet, and lack of physical activity, have all been impacted by the coronavirus disease 2019 (COVID-19) pandemic. This systematic review aims to explore the indirect effects of the first year of COVID-19 on obesity and its risk factors. A literature search of PubMed and EMBASE was performed from 1 January 2020 to 31 December 2020 to identify relevant studies pertaining to the first year of the COVID-19 pandemic (PROSPERO; CRD42020219433). All English-language studies on weight change and key obesity risk factors (psychosocial and socioeconomic health) during the COVID-19 pandemic were considered for inclusion. Of 805 full-text articles that were reviewed, 87 were included for analysis. The included studies observed increased food and alcohol consumption, increased sedentary time, worsening depressive symptoms, and increased financial stress. Overall, these results suggest that COVID-19 has exacerbated the current risk factors for obesity and is likely to worsen obesity rates in the near future. Future studies, and policy makers, will need to carefully consider their interdependency to develop effective interventions able to mitigate the obesity pandemic.

## Teaser

This is a systematic review demonstrating the impact of the first year of COVID-19 on key obesity risk factors, including diet, physical activity, depression, and financial stress.

## Introduction

With over 268 million infections and 5.2 million deaths worldwide (1), coronavirus disease 2019 (COVID-19) is one of the most serious infectious disease outbreaks in recent history. Even before the declaration of pandemic status by the WHO on 11 March 2020, many countries had begun to impose social-distancing measures (SDMs) in an attempt to reduce disease incidence. Understandably, the attention of scientists has focused on how to limit the short-term consequences of COVID-19, which were mitigated by SDMs until vaccines were released. As a result, the scientific community has prioritized the research on the determinants of mortality and morbidity of COVID-19 over the long-term implication of the virus and the necessary countermeasures, such as SDMs.

Obesity is defined by the WHO as abnormal or excessive fat accumulation that presents a risk to health, marked by a BMI (in kg/m^2^) >30, and has reached epidemic proportions (2). Statistics suggest that the prevalence continues to follow an increasing trajectory, with over 650 million adults having obesity in 2016 (3). Various models are attempting to predict the future burden of obesity, with projections ranging from 44% to >50% of the population (4, 5), although all agree that it is likely to encompass a significant proportion of the population. Many chronic illnesses are adversely affected by carrying excess body fat, with obesity being linked to cancers, cardiovascular disease, hypertension, and osteoarthritis, as well as a strong association with metabolic syndrome (6).

Among the factors that can increase the risk of obesity, some seem to play a more prominent role than others. For example, depression has repeatedly been shown to have bidirectional associations with obesity and overweight (7). The effect of depression on obesity is likely multifactorial, involving neuroendocrine disruption with a chronic state of elevated cortisol (8); lifestyle changes with reduced desire to exercise and increase in emotional eating (9); and, in some cases, the use of antidepressants (10). Socioeconomic status has long been linked inversely to body weight (11) and again is multifactorial with effects mediated through fewer opportunities for physical activity and healthy food and education and poorer mental health. Not only is low physical activity a risk factor for obesity but it is also an important modulator of risk conferred by excess weight (12), and so the potential effect of lockdowns on sedentary behavior may act as a multiplier for poor outcomes.

As a result of such health implications, obesity imposes a considerable economic burden, from the individual through national levels (13). In addition to direct effects on excess care needs, costs are also incurred through time off work, lower productivity at work, and associated disabilities. These costs have previously been estimated on a global scale to be 2.8% of global Gross Domestic Product (GDP) at US $2 trillion (14), since which time the proportion of the population having obesity has continued to rise.

The direct implications of COVID-19 on health and well-being are well-discussed elsewhere; what remains to be seen is whether this pandemic is exacerbating the growing obesity pandemic. A systematic review and meta-analysis by Bakaloudi et al. (15) suggest an overall global trend of weight gain during the first COVID-19 lockdown. To date, no studies have assessed the indirect impact of the COVID-19 pandemic, such as its SDMs, on obesity risk factors, that could explain this trend. Therefore, the objectives of this paper are to fill this gap by describing the effects of the COVID-19 pandemic and the needed countermeasures on obesity risk factors to explore underpinning mechanisms of the general trend of weight gain during the COVID-19 pandemic.

## Methods

### Search strategy and study selection

A literature search of PubMed and EMBASE was performed from 1 January 2020 to 31 December 2020 to identify relevant studies pertaining to the first year of the COVID-19 pandemic. The study was performed according to the Preferred Reporting Items for Systematic Reviews and Meta-Analyses (PRISMA) guidelines (16). The protocol details were registered prospectively on PROSPERO (CRD42020219433).

The following keywords were used in the search criteria: (“Sars-Cov-2” OR “covid-19”) AND (“quarantine” OR “lockdown” OR “BMI” OR “body mass index” OR “obese” OR “obesity” OR “overweight” OR “weight gain” OR “physical activity” OR “depression” OR “depressive symptoms” OR “redundancy” OR “redundant” OR “low income” OR “sedentary behaviour”). The search was limited to the English language, full-text availability, and human subjects. The abstracts of the resulting studies were manually searched to identify relevant studies, with NFD, CB, and TC applying inclusion/exclusion criteria to the full text to select the final studies.

### Inclusion and exclusion criteria

All English-language studies about weight change and key obesity risk factors (psychosocial and socioeconomic health) during the COVID-19 pandemic were considered for inclusion. Studies had to be comparative (baseline vs. during the pandemic) with cross-sectional and longitudinal studies considered. At least one of the following factors had to be included: *1*) weight (either anthropometry or self-report), *2*) dietary habit, *3*) physical activity, *4*) depressive symptoms, or *5*) financial status. In cases of depression, a validated depression measure had to be used [such as Patient Health Questionnaire (PHQ)-9] with any unvalidated questionnaires excluded (17–19). Qualitative studies, case reports, and reviews were excluded. Papers including pregnant women were also excluded due to the confounding effect of pregnancy over the outcomes of interest.

### Data extraction

Data extraction was performed independently by NFD, CB, and TC, with any ambiguity resolved via consensus. Each included study had the following extracted: *1*) study ID (author name and date), *2*) country, *3*) study type, *4*) sample size, *5*) sample characteristics (age, sex, and occupation of sample), *6*) assessment tool, and *7*) outcome.

### Data synthesis and quality assessment

Results were summarized via a narrative review; a quantitative synthesis was not attempted due to the heterogeneity of the samples and methodology between studies in the measurement of the relevant factors (e.g., depression). Study quality was assessed using a modified Newcastle Ottawa Scale (20), which was performed by NFD, CB, and TC, and any ambiguity was resolved via consensus (see **Supplemental Material**). The score used was based on the selection of the study sample using 4 criteria, the comparability of the outcome groups, and assessment of the outcome. The final score ranged from 0–10 points, with 0–4 considered unsatisfactory, 5–6 considered satisfactory, 7–8 considered good quality, and 9–10 points considered very good quality (20).

## Results

**FIGURE 1:**
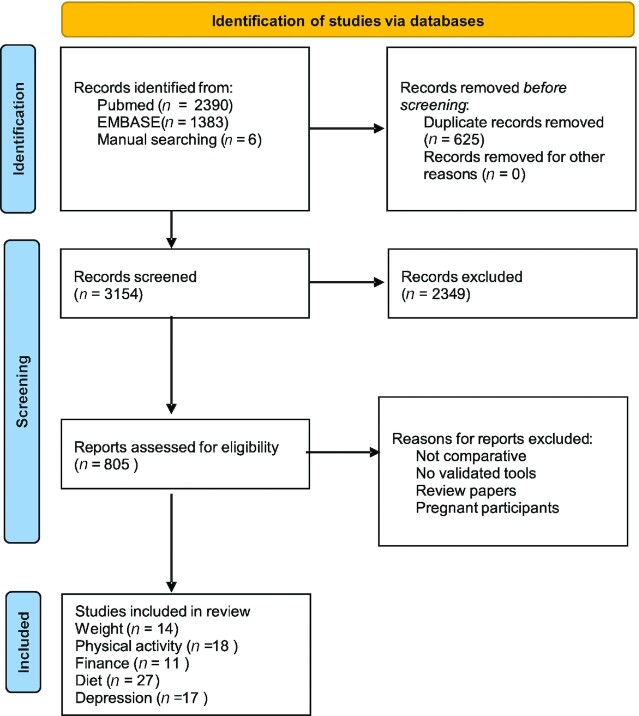
PRISMA flow diagram. PRISMA, Preferred Reporting Items for Systematic Review and Meta-Analysis.

The electronic search conducted identified 3773 studies (EMBASE: 1383; PubMed: 2390). After removing duplicates, 3154 studies were screened using a 2-step approach. First, the title and abstract of each paper were screened followed by a full-text screening if the inclusion and exclusion criteria were met. Based on screening the title and abstract, 805 (PubMed: 626; EMBASE: 179) potentially eligible studies were identified. Full-text screening resulted in a total of 87 studies that were included in the systematic review (**Figure 1**). A summary of the characteristics of included studies is presented in **Tables 1**–**5**.

**TABLE 1:** Characteristics of included studies investigating the relation between COVID-19 and weight^1^

Study ID	Country	Study type	No. of participants	Sample characteristics	Assessment tool	Outcome
Fernandez-Rio et al. 2020 (21)	Spain	Cross-sectional	4379	Age: 16–84 y Sex (F): 2671 (60.9%) Occupation/characteristics: General population	Self-reported weight	No weight changes: 52.88%Weight increase: 25.82%Weight decrease: 21.27%*P* value NR
de Luis Román et al. 2020 (30)	Spain	Cross-sectional	284	Age: 60.4 ± 10.8 y Sex (F): 211 (74.3%) Occupation/characteristics: Obese outpatients	Self-reported weight	36.3% reported weight gainIncrease in self-reported body weight was 1.62 ± 0.2 kg over 7 wk of confinement*P* value NR
Martínez-de-Quel et al. 2020 (31)	Spain	Longitudinal	161	Age: 35.0 ± 11.2 y Sex (F): 60 (37%) Occupation/characteristics: General population	Self-reported weight	Significant increase in weight (*P *= 0.012) during lockdown
López-Moreno et al. 2020 (33)	Spain	Cross-sectional	675	Age: 39.1 ± 12.9 y Sex (F): 472 (70%) Occupation/characteristics: General population	BMI	No significant change in BMI pre- and post-COVID-19 (*P *= 0.758)
Mason et al. 2020 (34)	USA	Longitudinal	1820	Age: 19.72 ± 0.46 y Sex (F): 1128 (62%) Occupation/characteristics: High school students	BMI	Overall significant increase in weight during COVID-19 relative to baseline (*P *< 0.001)
Yang et al. 2020 (29)	China	Cross-sectional	10,082	Age:High school students: 17 ± 1.2 y Undergraduate students: 20.6 ± 1.8 yGraduates: 24.6 ± 3.5 ySex: (F): 7229 (71.7%) Occupation/characteristics: Students	BMI	BMI significantly increased overall during COVID-19 (*P *< 0.001) in all subgroupsPrevalence of overweight/obesity significantly increased generally (*P *< 0.001) and in high school (*P *< 0 .01) and undergraduate students (*P *< 0 .001)
Jia et al. 2020 (32)	China	Cross-sectional	10,082	Age: 19.8 ± 2.3 y Sex (F):7229 (71.7%) Occupation/characteristics: Students	BMI	BMI significantly increased from 21.8 to 22.1 kg/m^2^ (*P *< 0.001)Significant increase in prevalence of overweight participants, (21.4% vs. 24.6%, *P *< 0.001) and obesity (10.5% vs. 12.6%, *P *< 0.001)
Pellegrini et al. 2020 (24)	Italy	Observational retrospective	150	Age: 47.9 ± 16 Sex (F): 116 (77.3%) Occupation/characteristics: Obesity outpatients	Self-reported weight	Significant increase in mean self-reported weight gain during COVID-19 ≈ 1.5 kg (*P* < 0.001)
Gallè et al. 2020 (25)	Italy	Cross-sectional	1430	Age: 22.9 ± 3.5 y Sex (F): 936 (65.5%) Characteristics: Italian undergraduate students	BMI	No significant change in BMI (*P *= 0.96) during COVID-19
Grabia et al. 2020 (28)	Poland	Cross-sectional	124	Age: 23 y (LQ-UQ 17–35) Sex (F): 103 (83%) Occupation/characteristics: Diabetic patients	Self-reported weight	Change in body mass(*P *< 0.001)Increased during COVID-19:49%≤5 kg: 31%>5 kg:11%No change: 28%Reduced: 30%
Sidor and Rzymski 2020 (23)	Poland	Cross-sectional	1097	Age: 27.7 ± 9.0 (18–71) y Sex (F): 1043 (95.1%) Occupation/characteristics: General population	Self-reported weight	Increase in weight: 29.9%Decrease in weight: 18.6% Those with high BMI at baseline experienced greater weight gain (*P *< 0.05), as did those older in age (*P *< 0.05)
Błaszczyk-Bębenek et al. 2020 (26)	Poland	Cross-sectional	312	Age: 41.12 ± 13.05 y Sex (F): 200 (64.1%) Occupation/characteristics: Age >18 y, not pregnant, no diseases requiring a specific diet	Self-reported weight	Statistically significant increase in weight during confinement (Δ 0.56 ± 2.43 kg; *P* < 0.0001)
Cheikh Ismail et al. 2020 (22)	Middle East and North Africa	Cross-sectional	2970	Age: 18+ y Sex (F): 2126 (71.6%) Occupation/characteristics: General population	Self-reported weight	No weight changes: 43.9%Weight increase: 30.3%Weight decrease: 16.9%*P* value NR Significant association between physical activity and reported change in weight (*P *< 0.001)
Pišot et al. 2020 (27)	9 European countries (Croatia, Italy, Serbia, Slovakia, Spain, Greece, Bosnia, and Kosovo)	Cross-sectional	4108	Age: 32.0 (13.2) y Sex (F): 2581 (62.8%) Occupation/characteristics: General population	Self-reported weight	Increase of 0.3 (±2.2) kg during COVID-19 pandemic measures (*P *< 0.0008) (*n *= 2208)

1 COVID-19, coronavirus disease 2019; NR, not reported; LQ-UQ, lower quartile-upper quartile; .

### Characteristics of included studies

Of the 87 studies included, 14 looked at the impact of COVID-19 on BMI directly (21–34), 18 looked at physical activity during the pandemic (31, 35–51), 11 looked at the financial impact (52–62), 27 at diet (23, 26, 33, 50, 61, 63–84), and 17 looked at depression (57, 85–100). None of the 87 studies investigated the link between the obesity risk factors and obesity itself. The majority of studies were conducted in the United States (*n *= 16), China (*n *= 13), Spain (*n *= 11), Poland (*n *= 6), and Italy (*n *= 7). The sample size ranged from 164,101 (100) to 18 (40) participants. In terms of quality assessment, there were a total of 2 unsatisfactory studies (51, 91), 36 satisfactory studies (21, 23, 25, 26, 28, 33, 36–38, 40, 41, 43, 44, 47, 48, 52–57, 59–64, 67, 68, 71, 77, 78, 81–83, 92), 42 good-quality studies (22, 24, 27, 29–32, 34, 39, 42, 45, 46, 49, 50, 57, 58, 61, 65, 66, 69, 70, 72–74, 76, 79, 80, 84–90, 93–98, 100), and 2 very good-quality studies (35, 99). Tables 1–5 show further details on the characteristics of the included studies.

**TABLE 2:** Characteristics of included studies investigating the relation between COVID-19 and physical activity^1^

Study ID	Country	Study type	Sample size	Sample characteristics	Assessment tool	Outcome
Wang et al. 2020 (35)	China	Longitudinal	3544	Age: 51.6 ± 8.9 y Sex (F): 1226 (34.6%) Occupation/characteristics: General population	Daily step counts recorded by the accelerometer sensor	Significant decrease in daily steps during COVID-19: reduced by 2678 (95% CI: 2582–2763)
Xiang et al. 2020 (51)	China	Longitudinal	2426	Age: 6–17 Sex (F): 1184 (48.8%) Occupation/characteristics: Children and adolescents (6–17 y)	WHO Global Physical Activity implantable cardioverter-defibrillators Questionnaire	Reduction in median time spent in physical activity (min/wk) during COVID-19: 540 vs. 105 (*P *< 0.001) Increase in prevalence of physically inactive students (21.3% vs. 65.6%), *P* value NRIncrease in screen time (min/wk) by +1730 min [or ∼30 h] per week on average (*P *< 0.001)
Sassone et al. 2020 (44)	Italy	Longitudinal	24	Age: 72 ± 10 y Sex (F): 7 (29%) Occupation/characteristics: Patients with implantable cardioverter-defibrillators	ICD-embedded accelerometric sensors	Significant reduction in physical activity during forced confinement (*P = *0.0001)
Tornaghi et al. 2020 (47)	Italy	Longitudinal	1568	Age: 15–18 y Sex: not stated Occupation/characteristics: High school students	IPAQ	No significant change in physical activity between during and pre-restriction or during and post-restriction COVID-19 rules Only highly active students increased their PA during and after the lockdown measures with respect to their baseline levels
Zheng et al. 2020 (45)	Hong Kong	Longitudinal (*n *= 70)Cross-sectional (*n *= 631)	631	Age: 21.2 ± 2.9 y Sex (M:F): 386 (61.2%) Occupation/characteristics: Young adults	IPAQ	Decrease in vigorous (*P* < 0.05) and moderate (*P* < 0.01) physical activity during COVID-19Significant decrease in walking during COVID-19 (*P *< 0.01) Significant increase in sedentary time during COVID-19 (*P *< 0.01)
Schmidt et al. 2020 (46)	Germany	Longitudinal	1711	Age: 4–17 y Sex (F): 852 (49.8%) Occupation/characteristics: 4–17-y-olds	Questionnaire	Increase of 0.44 active days per week (*P* < 0.01) during COVID-19 11.1% overall increase in adherence to WHO physical activity guidelinesScreen time guideline adherence decreased by 17.5% (*P *< 0.01)
Hanke et al. 2020 (48)	Germany	Longitudinal	248	Age: Females: 52.3 ± 13.7 y Males: 56.3 ± 13.7 y Sex (F): 89 (35.9%) Occupation/characteristics: Kidney transplant patients	Questionnaire	Significant decrease in sport (h/wk) during lockdown (*P *= 0.008) Significant increase in leisure activity^2^ (h/wk) (*P *< 0.001
Yang and Koenigstorfer 2020 (49)	USA	Longitudinal	431	Age: 39.1 ± 10.6 y Sex (F): 221 (51.3%) Occupation/characteristics: Healthy adults aged between 18 and 65 y old	IPAQ-SF	Significant decrease in moderate PA (*P *< 0.01), vigorous PA (*P* < 0.001) and PA in MET-min/wk (*P *< 0.01) during lockdown No significant change in sedentary time (*P* = 0.85) or walking (*P* = 0 .067)
Huckins et al. 2020 (37)	USA	Longitudinal	217	Age: 18–22 y Sex (F): 147 (67.8%) Occupation/characteristics: Undergraduate students	Mobile phone sensor data	Individuals were more sedentary during COVID-19 (*P *< 0.001)
Gallo et al. 2020 (50)	Australia	Longitudinal	2018 *n *= 174 (for PA 158)2019 *n *= 185 (for PA 177)2020 *n *= 150 (for PA 149)	Age: 19–27 y Sex (F):For physical activity: 2018: 97, 2019: 104, 2020: 84 Occupation/characteristics: Undergraduate students	Active Australia Survey	Males:Walking participation Significant reduction in 2020 combined with years 2018/2019, (*P < *0.05)Vigorous activity No difference between 2020 and years 2018/2019, (*P = *0.257) Females:Walking participationSignificant reduction in 2020 combined with years 2018/2019, (*P < *0.05)Vigorous activity No difference between 2020 and years 2018/2019 combined (*P = *0.245)
Hemphill et al. 2020 (36)	Canada	Longitudinal	109, of which 56 had longitudinal 2019 and 2020 data2019: *n *= 832020: *n *= 82	Age:2019: 13.0 ± 2.3 y 2020: 13.2 ± 2.3 ySex (F): 2019: 42% 2020: 48% Occupation/characteristics: Children with CHD aged 9–16 y	Step count data	Significant reduction in step count during lockdown (*P *< 0.001) During the early phase of the COVID-19 pandemic in Canada, children with CHD had a decline of 21–24% of their overall daily step counts
Bourdas and Zacharakis (2020) (38)	Greece	Longitudinal	8495	Age: 37.2 ± 0.2 y Sex (F): 5241 (61.7%) Occupation/characteristics: General population	Activity questionnaire	Overall physical activity decreased during lockdown measures (*P *< 0.05) Significant reduction (*P *< 0.05) in sporting activities
Munasinghe et al. (2020) (39)	Australia	Longitudinal	582	Age: 13–19 y Sex (F): 465 (79.9%) Occupation/characteristics: Adolescents	Questionnaire	Significant decrease in physical activity after physical-distancing measures
Muriel et al. (2020) (40)	Spain	Longitudinal	18	Age: 24.9 (2.8) y Sex (F): 0 (0%) Occupation/characteristics: Professional cyclists	Objective data collection—specialist software	Total training volume decreased by 33.9% during the lockdown (*P *< 0.01) Large reductions in best 5-min and best 20-min performances (*P *< 0.001)
Martínez-de-Quel et al. 2020 (31)	Spain	Longitudinal	161	Age: 35.0 ± 11.2 [19–65] y Sex (M:F): 60 (37%) Occupation/characteristics: General population	Minnesota Leisure Time Physical Activity Questionnaire (MLTPAQ)	Total physical activity significantly decreased during lockdown (*P < *0.001) Increase in number physically inactive during the pandemic (*P < *0.001)
Savage et al. (2020) (41)	UK	Longitudinal	214	Age: 20.0 y Sex (F): 154 (72%) Occupation/characteristics: Students	Questionnaire	Physical activity significantly decreased during the first 5 wk of lockdown (*P *< 0.01). Sedentary time significantly increased (*P *< 0.0001)
Vetrovsky et al. (2020) (42)	Czech Republic	Longitudinal	26	Age: 58.8 (9.8) y Sex (F): 8 (30.7%) Occupation/characteristics: Heart failure patients	Accelerometer	Significant decrease in daily step count during quarantine period (*P *< 0.001)
Zenic et al. (2020) (43)	Croatia	Longitudinal	823	Age: 16.5 ± 2.1 y Sex (F): NR Occupation/characteristics: Adolescents	Questionnaire	Physical activity levels significantly decreased during social distancing (*P* < 0.01).This was greater in urban than rural adolescents

1 CHD, congenital heart disease; COVID-19, coronavirus disease 2019; MET, metabolic equivalent of task; NR, not reported; PA, physical activity; ICD, implantable cardioverter-defibrillators; IPAQ-SF, Internatonal Physical Activity Questionnaire-Short form; .

2 Includes walks, bike rides, bicycle ergometer training, dancing, and bowling.

**TABLE 3:** Characteristics of included studies investigating the relation between COVID-19 and financial status^1^

Study ID	Country	Study type	Sample size	Sample characteristics	Assessment tool	Outcome
Evanoff et al. 2020 (52)	USA	Cross-sectional	5550	Age: not specified Sex (F): 4274 (77.3%) Occupation/characteristics: Benefits-eligible university faculty, staff, and postdoctoral scholars	Worse financial well-being due to COVID-19-related work or life changes, *n* (%)	Significant increase in worse financial well-being for 1732 (31.4%) *P* < 0.001
Wilson et al. 2020 (55)	USA	Cross-sectional	474	Age: median 40 (19–85) y Sex (F): 218 (46.4%)Occupation/characteristics: Currently employed adults	Questionnaire	Job insecurity:Not worried: 19.6%Slightly worried: 18.8% Some what worried: 23.2%Worried: 16.6%Very worried: 21.9% *P* value NR Financial concern over next 12 mo: Some degree of concern: 31.9%*P* value NR
Wanberg et al. 2020 (57)	USA	Longitudinal observational	1143	Age: 30–81 y Sex (F): 635 (55.6%) Occupation/characteristics: RAND American Life Panel, general population	Questionnaire	Laid off due to COVID-19: 40 (3.5%) Furloughed due to COVID-19: 32 (2.8%) *P* value NR
Donnelly and Farina 2020 (58)	USA	Cross-sectional	State-specific sample size ranging from 11,279 (Wyoming) to 77,811 (California)	Age: 44.4 ± 11.86 [18–65] y Sex (F): 61.76% Occupation/characteristics: General population	National survey	Reduction in household income after 13 March 2020: 45% of the analytic sample*P* value NR
McDowell et al. 2020 (59)	USA	Cross-sectional	2303	Age: 18–75 y Sex (F): 1520 (66%) Occupation/characteristics: Adults in employment before COVID-19	Working status	Lost employment due to pandemic: 13%*P* value NR
Almandoz et al. 2020 (61)	USA (Texas)	Cross-sectional	123	Age: 51.2 ± 13.0 ySex (F): 107 (87%)Occupation/characteristics: Adults with obesity	Survey/questionnaire	Lost job since COVID-19: 11 (9.6%)*P* value NR
García-Alvarez et al. 2020 (60)	Spain	Cross-sectional	21,207	Age: 39.7 ± 14.0 ySex (F): 14,768 (69.6%)Occupation/characteristics: General population	Questionnaire	Reduction in income due to COVID-19:Up to 25%: 2292 (10.8%)26–50%: 1367 (6.4%)51–100%: 1738 (8.2%)Income increase: 133 (0.6%)*P* value NR Job loss:Temporary or permanent lay off: 1871 (8.9%)Dismissal: 390 (1.9%)Forced vacation: 954 (4.5%)*P* value NR
Gualano et al. 2020 (62)	Italy	Cross-sectional	1515	Age: Median: 42 (IQR: 23) ySex (F): 973 (65.6%)Occupation/characteristics: General population	Questionnaire	Fear of losing employment:No: 543 (85.4%)Yes: 93 (14.6%)*P* value NR Income reduction:No: 46 (23.5%)Yes: 150 (76.5%)*P* value NR Job situation:Lay off: 98 (6.5%)Lost job: 18 (1.2%)*P* value NR
Song et al. 2020 (54)	China	Cross-sectional	709	Age: 35.35 ± 6.61 ySex (F): 526 (74.2%)Occupation/characteristics: Working adults, not infected	Questionnaire	Income change:Decrease: 244 (34.4%)No change: 436 (61.5%)Increase: 39 (4.1%)*P* value NR Some degree of worry about unemployment caused by COVID-19: 251 (35.5%)
Guo et al 2020 (53)	China	Cross-sectional	506	Age: 33.5 (14.0) Sex (F): 289 (57.1%) Occupation/characteristics: Patients with skin disease	Questionnaire	Decrease or loss of income in 317 (62.6%) during lockdown. P-value NR
Nienhuis and Lesser, 2020 (56)	Canada	Cross-sectional	1098	Age: 42 ± 15 Sex (F): 871 (79.3%) Occupation./characteristics: General population	Questionnaire	Change in work due to pandemic Men: 43% Women: 60% P-value NR Employment Status Post-COVID No change: 43.2% Reduced hours: 10% Remote work: 32.1% Loss of employment: 14.7% P-value NR

1 COVID-19, coronavirus disease 2019; NR, not reported.

**TABLE 4:** Characteristics of included studies investigating the relation between COVID-19 and diet^1^

Study ID	Country	Study type	Sample size	Sample characteristics	Assessment tool	Outcome
Alhusseini and Alqahtani, 2020 (80)	Saudi Arabia	Longitudinal observational	2706	Age: 18+ y Sex (F): 1466 (54.2%) Occupation/characteristics: General population	Dietary habit questionnaire	Increase in healthy food rating *(P < *0.05) Increased consumption of home-cooked meals (*P < *0.001) Increased quantity of food consumption (*P < *0.001)
Robinson et al. 2020 (81)	UK	Cross-sectional	2002	Age: 34.74 ± 12.3 y Sex (F): 1236 (62%) Occupation/characteristics: General population	Short 13-item food-frequency questionnaire (SFFQ)	Diet during COVID-19 relative to baseline:Better: 694 (35%)Same: 620 (31%)Worse: 688 (35%) 56% reported snacking more frequently*P* value NR Having a higher BMI was independently associated with lower diet quality (*P *< 0.01)
Buckland et al. 2020 (65)	UK	Cross-sectional	588	Age: 33.4 ± 12.6 y Sex (F): 403 (69%) Occupation/characteristics: General population	Questionnaire	Increased food consumption: 268 (48%)Increased meal amount: 173 (31%)*P* values NR
Do et al. 2020 (82)	Vietnam	Cross-sectional	5209	Age:21–40 y: 4304 (82.6%)41–60 y: 905 (17.4%) Sex (F): 3495 (67.1%) Occupation/characteristics: Health care workers	Online survey	Dietary change compared with pre-pandemic:Unchanged or healthier: 5042 (96.8%)Lesshealthy: 167 (3.2%)*P* value NR
Carroll et al. 2020 (84)	Canada	Cross-sectional data (from longitudinal study)	361 parents from 254 families	Age:Mothers 39.4 (SD 5.5) yFathers 37.5 (SD 4.8) y Children 5.7 (SD 2.0) y Sex: (F): 235 (65%) Occupation/characteristics: Families with young children	Food questionnaire	Eating more food since confinement (mothers, 57%; fathers, 46%; children, 42%) More snack foods (mothers, 67%; fathers, 59%; children, 55%)*P* value NR
Huber et al. 2020 (63)	Germany	Cross-sectional	1964	Age: 23.3 ± 4.0 y Sex (F): 1404 (71.5%) Occupation/characteristics: University students	Questionnaire	Overall food intake during lockdown:Increased: 31.2%Decreased: 16.8%*P* value NR Increase in food intake was mainly triggered by consumption of bread (increased in 46.8%) and confectionery (increased in 64.4%).*P* value NR
Visser et al. 2020 (64)	Netherlands	Longitudinal cohort	1119	Age: 74 ± 7 y Sex (F): 593 (52.8%) Occupation/characteristics: Dutch older adults	Questionnaire	Change in eating habits during pandemic:Eating less than normal: 12.1%*P = *0.003Eating too little or losing weight: 6.6%*P = *0.260Snacking more: 32.4% *P <* 0.001Skipping warm meals: 9.1%*P = *0.003
López-Moreno et al. 2020 (33)	Spain	Cross-sectional	675	Age: 39.1 ± 12.9 y Sex (F): 472 (70%) Characteristics: General public	Questionnaire	Overall worsening of diet: 112 (16.2%)Increased food intake: 19.6%Increased purchase of snacks: 39% Increased purchase of processed foods: 25%*P* value NR Overall improvement of diet: 266 (38.4%)Decreased food intake: 33.3%*P* value NR
Rodríguez-Pérez et al. 2020 (77)	Spain	Cross-sectional	7514	Age: ≤20 y: 22921–35 y: 2558 36–50 y: 237151–65 y: 1928≥65 y: 428 Sex (F): 5305 (70.6%) Occupation/characteristics: General population	Mediterranean Diet Adherence Screener (MEDAS)	Increased adherence to Mediterranean diet (*P < *0.001) Reduced alcohol intake *(P < *0.001) Self-reported “not eating more” during confinement: 63.7% (*P < *0.001)
Sánchez-Sánchez et al. 2020 (72)	Spain	Cross-sectional	1065	Age: 38.7 ± 12.4 y Sex (F): 775 (72.8%) Occupation/characteristics: General population	Mediterranean Diet PREDIMED questionnaire	Increased adherence to Mediterranean diet (*P = *0.004) Significant increase in daily portions of vegetables, olive oil, fruit, red meat, sugary/carbonated beverages (*P < *0.05) Significant increase in proportion drinking wine ≥7×/wk (*P < *0.001)
Ruiz-Roso et al. 2020 (69)	Spain (Madrid)	Cross-sectional	72	Age: 41.12 ± 13.05 ySex (F): 46 (64.1%) Occupation/characteristics: Cohort of adults with T2D(1) Between the age of 40 and 80 y, (2) BMI ≥25 and <40 kg/m^2^	Phone interview	Snacking:Increased sugary food servings≥5 times/wk (2.9% vs. 5.7%)Increased snacking≥4 times/wk (5.7% vs. 12.9%) Significant increase in vegetable consumption (*P < *0.0001)
Di Renzo et al. 2020 (66)	Italy	Cross-sectional	3533	Age: 40.03 ± 13.53 [12–86] y Sex (F): 848 (24%) Occupation/characteristics: General population	Mediterranean Diet Adherence Screener (MEDAS)	Healthier diet (fruit, vegetables, nuts and legumes): 37.4%Unhealthier diet: 35.8%*P* value NR Significant decrease in junk food consumption (*P = *0.002)
Pietrobelli et al. 2020 (67)	Italy	Longitudinal	41	Age: 13.0 ± 3.1 y Sex (F): 19 (46%) Occupation/characteristics: Children and adolescents with obesity	Interview and questionnaire	Increased number of daily meals (*P < *0.001) Increased fruit intake (*P = *0.055); no change in vegetable intake Increase in potato chips, red meat, and sugary drink intake (*P = *0.005)
Almandoz et al. 2020 (61)	USA (Texas)	Cross-sectional	123	Age: 51.2 ± 13.0 y Sex (F): 107 (87%) Occupation/characteristics: Adults with obesity	Survey/questionnaire	Dietary changes during pandemic:Stress eating: 61.2%Cooking more often: 63.8%Food behaviors:Reported healthy eating to be more challenging during pandemic: 61.2%Skipping meals when not food insecure: 12.1%*P* value NR
Knell et al. 2020 (73)	USA	Cross-sectional	1809	Age: 18+ y Sex (F): 1220 (67.4%) Occupation/characteristics: General population	Alcohol questionnaire	Significant increase in alcohol consumption (*P < *0.01)
Błaszczyk-Bębenek et al. 2020 (26)	Poland	Cross-sectional	312	Age: 41.12 ± 13.05 y Sex (F): 200 (64.1%) Occupation/characteristics: General population	Dietary Habits and Nutrition Beliefs Questionnaire	Significant increase in number of meals consumed and snacking (*P < *0.0001) Significant increase in alcohol (*P = *0.0031) Significant decrease in takeaways and fast food (*P < *0.0001) Significant decrease in energy drink consumption (*P = *0.015)
Sidor and Rzymski 2020 (23)	Poland	Cross-sectional	1097	Age: 27.7 ± 9.0 [18–71] y Sex (F):1043 (95.1%) Occupation/characteristics: General population	Questionnaire	Dietary changes during pandemic:Eating more: 43.5%More frequent snacking: 51.8%Cooking more often: 62.3%*P* value NR Alcohol intake changes:Increase: 14.6%No change: 77%Unsure: 8.3%*P* value NR
Górnicka et al. 2020 (68)	Poland	Cross-sectional	2381	Age:≤30y: 70030–39 y: 106740–49 y: 30650–59 y: 160 Sex (F): 2138 (89%) Occupation/characteristics: Over 18 y, not pregnant or lactating/breastfeeding	Questionnaire	Increase in unhealthy eating (*P < *0.001) Increase in confectionary and alcohol (*P < *0.001) Positive dietary changes during pandemic:Increased water intake (*P < *0.001) Decreased fast-food intake (*P < *0.001) Increased consumption of homemade meals (*P < *0.001)
Yan et al. 2020 (78)	China	Cross-sectional	9016	Age:18–80 y Sex (F): 5177 (57.4%) Occupation/characteristics: General population	Alcohol question	Significant increase in alcohol consumption (*P < *0.001) 54% diabetic and 10.2% nondiabetic participants reported significant increases in drinking
Wang et al. 2020 (70)	China	Cross-sectional	2289	Age: 17.8 ± 12 y Sex (F): 1113 (49%) Occupation/characteristics: Healthy Chinese adults	Questionnaire adapted from online nutritional survey of Guangdong Nutrition Society and Sun Yat-sen University	Daily eating frequency:Reduced: 23.1% No change: 60%Increased: 17.3% Food behavior changes:Appetite unchanged: 71.4%Healthier diet: 23%More vegetables,fruits and milk: >30% Increased snacking: ∼30%*P* value NR
Elran-Barak and Mozeikov 2020 (71)	Israel	Cross-sectional	315	Age: 18+ y Sex (F): 178 (59.5%) Occupation/characteristics: Israelis with a variety of chronic conditions	Questionnaire	Overall food consumption:Much more than before: 19.7%A little more than before: 30.5%Same as before: 40.0%A little less than before: 7.0%Much less than before: 2.9%*P* value NR No significant change in fruit consumption (*P = *0.060); decrease in vegetable consumption (*P = *0.008)
Gallo et al. 2020 (50)	Australia	Cross-sectional	2018 *n *= 174 (for diet 166)2019*n *= 185 (for diet 159)2020*n *= 150 (for diet 146)	Age: 19–27 y Sex (F):2018: 1012019: 962020: 82 Occupation/characteristics: Third-year biomedical practical students from University of Queensland in 2018, 2019, 2020	Automated self-administered dietary assessment tool	Total energy intake over 24 h (females): No significant change between 2019/2020 (*P = *0.067); significant increase between 2018 and 2020 (*P < *0.05) Total energy intake over 24 h (males): No significant difference
Husain and Ashkanani 2020 (74)	Kuwait	Cross-sectional	415	Age: 38.47 ± 12.73 y Sex (F): 285 (68.7%) Occupation/characteristics: General population	Questionnaire	Significantly increased snacking (*P* = 0.006), more late-night snacks (*P* < 0.001). Main meal was significantly more likely to be freshly made (*P* = 0.001), with reductions in fast-food consumption (*P* < 0.001). Decreased frequency of seafood consumption; no change in beverage consumption
Steele et al. 2020 (75)	Brazil	Longitudinal	10,116	Age:18–39 y: 5174 (51.1%) 40–59 y: 4034 (39.9%)≥60 y: 908 (9.0%) Sex (F): 7895 (78.0%) Occupation/characteristics: Adults >18 y, NutriNet Brasil Cohort	Adaptation of an instrument developed by the authors for the Ministry of Health Surveillance of Risk and Protective Factors for Chronic Diseases by Telephone Survey	Dietary behavior changes during pandemic:Increased consumption of vegetables and fruits (*P < *0.05) Increased consumption of beans/legumes (*P < *0.05)
Malta et al. 2020 (76)	Brazil	Cross-sectional	45,161	Age: 18+ y Sex (F): 24,206 (53.6%) Occupation/characteristics: General population	Covid Behavior Survey	Alcohol consumption:Increased: 17.6%*P* value NR Healthy food consumption:Decreased regular consumption of vegetables (37.3% vs. 33%) Unhealthy food consumption ≥2 d/wk:Increase in frozen food intake (10.0% vs. 14.6%).Increase in savory snacks:(9.5% vs. 13.2%).Increased consumption of chocolate/desserts (41.3% vs. 47.1%)*P* value NR
Ruiz-Roso et al. 2020 (79)	Italy, Spain, Chile, Colombia, and Brazil	Cross-sectional	820	Age: 15 (10–19) y Sex (F): 501 (61.1%) Occupation/characteristics: Adolescents between 10–19 y	Online questionnaire	Legumes, vegetables, and fruit intakes were significantly increased (*P* < 0.05); reduced fast-food consumption (*P*< 0.0001) Increased intake of fried foods and sweet foods (*P* < 0.001)
Ammar et al. 2020 (83)	Asia (36%), Africa (40%), Europe (21%), and other (3%)	Cross-sectional survey	1047	Age: 18+ y Sex (F): 563 (53.8%) Occupation/characteristics: General population	Short Diet Behaviour Questionnaire for Lockdowns (SDBQ-L)	Increase in self-reported unhealthy eating (*P < *0.001) Increased uncontrolled eating *(P < *0.001) Increased snacking (*P < *0.05)

1 COVID-19, coronavirus disease 2019; NR, not reported; PREDIMED, Prevención con Dieta Mediterránea.

**TABLE 5:** Characteristics of included studies investigating the relation between COVID-19 and depression^1^

Study ID	Country	Study type	Sample size	Sample characteristics	Assessment tool	Outcome
Chen et al. 2020 (85)	Hong Kong	Longitudinal	543 (completed both baseline and follow-up)	Age: 10.88 ± 0.72 y Sex (F): 273 (51%) Occupation/characteristics: Schoolchildren	DASS-21	Significant increase in DASS-21 during COVID-19 (*P < *0.001)
Ettman et al. 2020 (93)	USA	Cross-sectional w/comparison to NHANES data 2017–2018	1441 during pandemic, 5065 pre-pandemic	Age: 18+ y Sex (F):Baseline: 2588 (51.4%)Post-pandemic:718 (51.9%) Occupation/characteristics: General population	PHQ-9	More than 3-fold increase in depression symptoms during COVID-19*P* value NR Prevalence of depressive symptoms baseline vs. during pandemic:Mild depressive symptoms: 1.5-fold higherModerate depressive symptoms: 2.6-fold higherModerately severe depressive symptoms: 3.7-fold higherSevere depressive symptoms: 7.5 fold higher*P* value NR
Kannampallil et al. 2020 (94)	USA	Cross-sectional	393	Age: Not included Sex (F): 218 (55.5%) Occupation/characteristics: Physician trainees	DASS-21	No significant difference in DASS-21 score between those exposed to COVID and those not (*P = *0.70)
Coughenour et al. 2020 (86)	USA	Longitudinal	194	Age: 25.11 (SD 7.84) y Sex (F): 140 (72.2%) Occupation/characteristics: College students	PHQ-9	Significant increase in PHQ-9 depression score after stay-at-home order (*P < *0.01)
Flentje et al. 2020 (92)	USA	Longitudinal	2288	Age: 36.9 ± 14.7 y Sex (F): 1428 (63.0%) Occupation/characteristic: LGBT population	PHQ-9	Significant increase in PHQ-9 depression score in the total population during COVID-19 (*P *< 0 .001) Significant decrease in PHQ-9 depression score in those with a positive baseline screen (*P *< 0.001) Significant increase in PHQ-9 depression score in those with a negative baseline screen (*P *< 0 .001)
Wanberg et al. 2020 (57)	USA	Longitudinal	1143	Age: 30–81 y Sex (F): 635 (55.6%) Occupation/characteristics: RAND American Life Panel, general population	PHQ-8	Significant increase in depressive symptoms during the pandemic (*P = *0.01)
Xiang et al. 2020 (95)	China (Shanghai)	Longitudinal	2427	Age: 6–17 y Sex (F): 1185 (49%) Occupation/characteristics: School-age children	Children's Depression Inventory–Short Form (CDI‐S)	Significant decrease in CDI-S score, 4.19 baseline vs. 3.90 during school closure (*P < *0.01) Therefore. no evidence of increased depressive symptoms among students after a 2‐mo school closure
Liu et al. 2020 (96)	China	Cross-sectional	2126	Age: 16+ y Sex (F): 2077 (97.7%) Occupation/characteristics: Obstetrician: 770; midwife: 1356	PHQ-9	Significant increase in PHQ-9 score during COVID-19 (*P < *0.001) Those with direct contact with COVID-19 more likely to have severe depression (*P < *0.05)
Cai et al. 2020 (98)	China	Longitudinal study	1330: 709 (53.3%) from the outbreak period and 621 (46.7%) from the stable period	Age: 18+ y Sex (F):Peak: 684 (96.5%) Stable: 605 (97.4%) Occupation/characteristics: Nurses	PHQ-9	Significant increase in mean PHQ-9 score during the pandemic (4.67 vs. 5.59, *P < *0.001) During the outbreak, nurses had significantly higher proportions of depressive symptoms (*P < *0.001) Depression significantly higher in those on the frontline (*P < *0.05)
Li et al. 2020 (100)	China	Longitudinal	During outbreak (T1) (*n *= 164,101)During remission (T2) (*n *= 148,343)	Age: Not specified Sex (F):During outbreak: 103,645 (63.2%)During remission: 92,859 (62.6%) Occupation/characteristics: College students	PHQ-9	Increase in PHQ-9 depression score during remission (3.66 vs. 3.95)*P* value NR Significant increase in prevalence of depression (PHQ-9 score >9) during remission (*P < *0.001) Depression more likely in seniors and those who consumed alcohol *(P < *0.001)
Li et al. 2020 (91)	China	Longitudinal	385	Age: median: 25 (IQR: 23–28) y Sex (F): 247 (64%) Occupation/characteristics: Physicians from 12 Shanghai hospitals who enrolled in the prospective Intern Health Study in August 2019	PHQ-9	Significant increase in depressive symptoms from T1 (pre-pandemic) to T2 (during pandemic) 95% CI: 0.08, 1.14*P = *0 .02
Quittkat et al. 2020 (97)	Germany	Cross-sectional	586	Age: 34.06 ± 13.45 y Sex (F): 470 (80%) Occupation/characteristics: Pre-existing depression	DASS-D	Depression compared with pre-pandemic:Considerable improvement: 48 (8.19%)Slight improvement: 113 (19.28%) No change: 88 (15.02%) Slight worsening: 218 (37.2%)Considerable worsening: 119 (20.3%)*P* value NR
Thombs et al. 2020 (99)	Canada, France, UK, US	Longitudinal study	388	Age: 56.9 (SD 12.6) y Sex (F): 343 (88.5%) Occupation/characteristics: Systemic sclerosis patients	PHQ-8	Changes in depressive symptoms were minimal (reduction of 0.3 points, 95% CI: -0.7, 0.2) during pandemic*P* value NR
Elmer et al. 2020 (87)	Switzerland	Longitudinal	*n* = 212 (who experienced the crisis)*n* = 54 (earlier cohort who did not)	Age: Unspecified Sex (F):Current year, Major I (*n *= 70) 33.7% Current year, Major II (*n *= 142) 15.3% Previous year, Major I (*n* = 54) 38.9% Occupation/characteristics: Undergraduate students	CES-D	Students became significantly more depressed during the pandemic (mean_diff_ = 4.44, *P* < 0 .001) No significant difference between Majors
Pieh et al. 2020 (88)	Austria	Cross-sectional (compared to Austrian Health Interview Survey 2014)	1005	Age:18+ y Sex (F): 530 (52.7%) Occupation/characteristics: General population	PHQ-8	Significant increase in PHQ-8 depression score during pandemic (2.5 vs. 5.9, *P < *0.001)
Munk et al. 2020 (89)	Germany	Cross-sectional	949	Age: 28.9 ± 10.8 y Sex (F): 754 (79.5%) Occupation/characteristics: Recruited via Justus-Liebig University e-mail, and social media	BDI	Clinically depressive symptoms:Baseline: 7.7% depression rate )During pandemic: 35.3% (BDI score >13)*P* value NR
Schmitz et al. 2020 (90)	Canada	Cross-sectional	1607 (Quebec sample) 52,996 (CCHS sample^2^)	Age: 18+ y Sex (F) CCHC: 51.2% Quebec: 51.3% Occupation/characteristics: General population	PHQ-8 (compared to PHQ-9 in CCHS)	Increase in score >10 in PHQ-8 during pandemic (6.8% vs. 19.2%) Reported depressive symptoms: Baseline: Males: 5% Females: 9%During pandemic: Males: 17% Females: 22%*P* value NR

1 BDI, Beck Depression Inventory; CCHS, Canadian Community Health Survey; CES-D, Center for Epidemiologic Studies–Depression; COVID-19, coronavirus disease 2019; DASS, Depression, Anxiety and Stress Scale; LGBT, lesbian, gay, bisexual, transgender; NR, not reported; PHQ, Patient Health Questionnaire.

2 Baseline data from the 2015/2016 CCHS.

### Relation between COVID-19 and weight

A summary of the weight changes reported during COVID-19 is shown in Table 1. A total of 14 studies looking at the impact of COVID-19 on weight directly were included (21–30, 32–34, 75). Overall, there was a general trend of weight gain during the pandemic, with 12 studies reporting this. Although 3 studies included student populations (29, 32, 34) and 1 study looked at diabetic patients (28), the majority of the studies focused on the general population (22–24, 26, 27, 31). Different results were seen in Spain, in which 1 study reported no change in weight in the Spanish general population (33). This study by López-Moreno et al. (33) focused on BMI change, whereas the other 3 studies (21, 30, 31) used self-reported weight.

### Obesity risk factors and COVID-19

#### Relation between COVID-19 and physical activity

A summary of the changes in physical activity during the first year of COVID-19 is shown in Table 2. A total of 18 studies were included that looked at the relation between COVID-19 and changes in physical activity and sedentary behavior (24, 36, 45–52, 37–44). All of the 18 studies were longitudinal and used self-reported measurements, except for Wang et al. (35), who used an accelerometer sensor to record daily step counts. A total of 16 studies reported a reduction in physical activity during COVID-19, with 1 study showing an increase in activity (46) and 1 showing no change at all (40). A study in German schoolchildren aged between 4 and 17 y found an increase in active days per week, with an 11.1% increase in adherence to WHO physical activity guidelines (46). A study of high school students found no significant increment in physical activity during COVID-19 compared with the pre-restriction baseline; however, highly active students increased their activity levels relative to baseline (47).

#### Relation between COVID-19 and diet

Twenty-seven studies were included that investigated the impact of COVID-19 on dietary patterns, as summarized in Table 4.

##### Favorable changes in dietary behavior

A total of 5 studies reported an increase in home-cooked meals during the pandemic (23, 61, 68, 74, 80). Three studies reported an overall reduction in the frequency of fast food (26, 74, 79). Of the studies looking at alcohol consumption, only 1 study found a decrease in alcohol consumption during the pandemic in the Spanish general population (77). This decline in alcohol was correlated with higher adherence to the Mediterranean diet.

A cross-sectional study of the general population in Italy found an increase in the consumption of fruit, vegetables, nuts, and legumes and a significant decrease in junk food consumption (66). Second, a Spanish cross-sectional study focusing on patients with type 2 diabetes found a significant increase in vegetable consumption during the pandemic (69). Third, a study looking at healthy Chinese adults found an increase in vegetable, fruit, and milk consumption (70) relative to before the pandemic. The last change reported by the studies was a reduction in overall food consumption during the pandemic (26, 82). A longitudinal study of adults older than 62 y in the Netherlands found that 12% of the sample were eating less than usual. However, this change in dietary habits was not reflected by a statistically significant reduction in weight (64).

##### Unfavorable changes in dietary behavior

A total of 7 studies reported an increase in alcohol consumption (23, 26, 68, 72, 73, 76, 78). Three of the studies were in the Polish general population (23, 26, 68), with the remainder reporting from Spain (72), the United States (73), China (78), and Brazil (76). A total of 10 studies found an increase in the quantity of food consumed during COVID-19 (23, 26, 50, 63, 65, 67, 71, 80, 83, 84). In particular, the most common change during the pandemic was an increase in snacking frequency, which was reported in 11 studies that included patients from a wide range of geographical areas ranging from Europe to Asia and including North America (23, 26, 33, 61, 64, 69, 70, 74, 81, 83, 84).

#### Relation between COVID-19 and socioeconomic status

Eleven studies were included in this review that investigated the impact of COVID-19 on financial status, as summarized in Table 3. Out of these studies, one reported a statistically significant worsening of financial well-being among 5550 benefits-eligible university staff (94). The remaining studies did not report a *P* value or 95% CI but reported a detrimental impact of COVID-19 on financial status, resulting in either reduced income (53, 54, 58, 60, 62) or job loss (56, 57, 59–62). Two of the papers showed that COVID-19 resulted in alarming the participant and increasing their fear of job insecurity (55, 62), with Wilson et al. (55) reporting that 31.9% of participants had financial fears during the pandemic and only 19.6% of the sample had no concerns at all.

#### Relation between COVID-19 and depression

Seventeen of the studies included in this review investigated the relation between COVID-19 and depression, as summarized in Table 5. Only validated depression scales were used, of which 3 studies used the Depression, Anxiety and Stress Scale (DASS) (85, 94, 97), 11 studies used the PHQ (57, 86, 88, 90–93, 96, 98–100), 1 study used the Children's Depression Inventory–Short Form (CDI‐S) (51), 1 study used the Center for Epidemiologic Studies–Depression (CES-D) (101), and 1 study used the Beck Depression Inventory (BDI) (89).

Ten studies reported a statistically significant increase in depressive symptoms during the pandemic (59, 89, 91, 93–96, 99–101). Two of the studies looked at the general population in the United States (57) and Austria (88). Three of these studies investigated clinical staff including obstetricians and midwives (96), nurses (98), and physicians (91). Four studies looked at a younger cohort of participants including schoolchildren (85) and students (86, 87, 100). Finally, one of the studies looked at the impact of COVID-19 on the LGBT (lesbian, gay, bisexual, transgender) population in the United States and found a significant increase in depressive symptoms, particularly in those with a negative baseline screen (92). Although the *P* value was not reported in 7 studies (89, 90, 93, 94, 97, 99, 100), 6 of them reported a trend of increased depression scores during COVID-19 (89, 90, 93, 97, 99, 100). Only 1 study found no increase in depressive symptoms during COVID-19 and looked at US physician trainees (94).

## Discussion

This systematic review of over 350,000 participants from across the globe attempted to describe the indirect impact that the SDMs due to the COVID-19 pandemic had on population body weight by altering the most important risk factors—namely, diet, physical activity, mental health, and financial status. Although the impact of the countermeasures used to curb the COVID-19 pandemic was evident on obesity risk factors, none of the studies included in our research explored the direct impact of the risk factors on obesity itself.

The general trend seen in included studies was a worsening in the obesity risk factors. There were, however, notable exceptions. A German study in schoolchildren found an improvement in physical activity (46) due to recreational sporting activities. This discrepancy is likely due to contextual factors, such as how stringent the SDMs were in the specific countries. For example, in China, outdoor physical activity was banned during the first wave of COVID-19 (46).

Differences were also seen in dietary changes, with some studies showing an improvement in diet. However, those studies showing improvements in diet were looking at very different subgroups of the population (66, 69, 70), including the elderly or those with underlying medical conditions. The age of participants appears to have an impact, with the largest sample-size studies (25, 34) showing a significant weight increase in those under age 25. The same was seen in a US sample of students (35). This may reflect the widespread reduction in activity and greater sedentary time in this group of people across multiple nations (36, 38, 43, 46, 50). It may also suggest a disproportionate impact of SDMs on the younger population. However, a comparable group of undergraduate students in Italy (30) did not show an increase in weight, which suggests a potential cultural role.

The proximity to COVID-19 exposure may have played a role in the likelihood to report increased stress or depressive symptoms, as was seen in several cohorts of health care workers (89, 91, 99). These studies did, however, tend to occur earlier in the course of SDMs, which could also have played a role as uncertainty was at its greatest early on in the pandemic.

The COVID-19 pandemic, and its related SDMs, led to a worsening of obesity risk factors in the majority of studies—albeit some beneficial effects were observed in the dieting domain, such as higher consumption of home-cooked meals and healthy food (e.g., vegetables). On the other hand, the overall food and alcohol consumption showed an increasing trend, which could have been either the result or the cause of poorer mental health (102).

An unavoidable consequence of the SDMs and, in the most extreme cases, of the national lockdowns was financial hardship and job loss. A large body of evidence suggests that financial stress is linked to mental illness, which, then, could have fueled the obesity risk factors mentioned previously (103). Another element adding an extra level of complexity is the bidirectional relation between financial hardship, mental illness, and the other obesity risk factors, which makes it problematic to draw a conclusion on which is the leading factor during stressful circumstances, such as a pandemic.

There are several notable papers in the literature that have been published during the writing of this report, which go some way to supporting our conclusions. Jia (104), Browne et al. (105), and Knebush et al. (106) all discuss similar findings with the interaction between the coronavirus pandemic and obesogenic risk factors. Jia (104) highlights the multifactorial impact of the pandemic on the obesogenic environment in adolescents, including increased sedentary time and dietary changes. Upstream factors, such as changes in food environments and interaction with the built environment, might help to explain some of our findings; however, as noted by Jia, more modern measurement techniques are needed to better quantify this. An important issue raised is the difficulty in following up cohorts during periods of lockdown and how this will affect future data trends.

Browne et al. (105) also considered the change in the obesogenic environment affecting children during the COVID-19 pandemic. Increased stress has arisen from changes to home and school environments, in concert with less engagement in physical activity and increased familial financial stress. As we have found the case to be in adults, this review suggests that COVID-19 has exacerbated the obesity pandemic in children. An additional consideration in this paper was the deleterious impact of weight stigma, which can further increase the psychological and physical sequelae of obesity.

Knebush et al. (106) again noted similar patterns of reduced physical activity, increased screen time, and dietary changes. School closures have had a marked impact on each of these risk factors at critical points in a child's development.

These papers all highlight a similar pattern of an increasingly obesogenic environment that children have been subjected to during multiple SDMs throughout the pandemic. Of interest will be the effect of this in years to come as these children become adults, perpetuating the trend for increasing weight.

A *BMJ* feature (107) highlights the voice of Christina Marriott, chief executive of the Royal Society of Public Health, on the topic of obesity in the COVID-19 pandemic, who states that there has not been sufficient action to address the root causes of obesity. For this to happen, the complex relation between the obesity risk factors should be explored in quantitative studies. Our review acts to emphasize the areas in which further data are required. In addition to this, there is a clear need for cost-effective policies able to mitigate the impact on obesity of stressful circumstances, such as a pandemic.

Our research is the first to attempt to summarize the multifactorial implications that the SDMs due to the COVID-19 pandemic had on obesity. A very broad search strategy was adopted to capture as thorough a picture as possible, aiming to include papers noting an association between COVID-19 SDMs, obesity, and risk factors together. None of the studies included in our research investigated the link between *1*) SDMs, *2*) obesity risk factors, and *3*) obesity itself. The absence of studies linking (*1*) to (*2*) and, thus (*3*), led us to focus our review on the impact of SDMs on obesity risk factors. As a consequence, our review cannot provide a conclusion on which elements have driven the increment in BMI during the COVID-19 pandemic (15). While this is the most important weakness of our study, our broad literature review allowed us to identify the studies on the effects of the pandemic on obesity and its risk factors.

Although our contribution is not sufficient to draw a conclusion, it represents a necessary step to develop new studies able to determine the key drivers of obesity in stressful circumstances, such as a pandemic. In addition to the absence of evidence necessary to draw a conclusion, many of the included studies focused either on self-reported body weight or BMI. Although these are widely used and validated measures of identifying individuals at risk of overweight or obesity, they do not account for factors that more reliably and objectively link to health outcomes, such as total body fat percentage.

Another limitation of our review is the high proportion of cross-sectional studies, which makes it problematic to establish a causal link. Likewise, the high heterogeneity in methodology, samples, and socioeconomic characteristics made comparisons difficult. Many of the studies had a significantly higher response rate in females, which may somewhat limit the application of our conclusions to the general population. Several studies also focused on specific groups, many of which used health care workers or students. Once again, this may limit the generalizability of our conclusions.

These limitations are acknowledged in our quality assessment of the included studies. However, given the circumstances in which many of these studies were carried out, amid national lockdowns, in-person data collection was often unfeasible and so the majority of studies were affected by this measurement issue.

While this review does not provide a conclusive answer on the driver of obesity during the COVID-19 pandemic, it provides useful information to direct future research aiming at strengthening the link between stressful circumstances and a rise in risk factors for obesity and weight gain. This is important as establishing a link enables us to effectively target the risk factors in preventative public health measures. There is a need for longitudinal studies to elucidate the nature of the association.

## Supplementary Material

nzac011_Supplemental_File

## Notes

The authors report no funding received for this work.

Author disclosures: The authors report no conflicts of interest.

Abbreviations used: COVID-19, coronavirus disease 2019; PHQ, Patient Health Questionnaire; SDM, social-distancing measure.
