# Supplementary material for: A Systematic Review of the Impact of the First Year of COVID-19 on Obesity Risk Factors: A Pandemic Fueling a Pandemic?
Source: Curr Dev Nutr. 2022 Apr 8;6(4):nzac011. doi: 10.1093/cdn/nzac011 (PMC8989548; doi:10.1093/cdn/nzac011)
Supplement: nzac011_Supplemental_File [file nzac011_supplemental_file.docx]

 A systematic review of the impact of the first year of COVID-19 on obesity: A pandemic fuelling a pandemic?

Natasha Daniels et al.

Online Supplementary Material

| **Author** | **Representativeness of sample** | **Sample size** | **Non-respondents** | **Ascertainment of the exposure** | **Comparability** | **Assessment of the outcome** | **Statistics** | **Total 'stars'** |
| --- | --- | --- | --- | --- | --- | --- | --- | --- |
| **Xiang M et al. 2020 (51)** | 0 | 0 | 0 | 2 | 0 | 1 | 1 | **4** |
| **Li W et al. 2020 (91)** | 0 | 0 | 0 | 2 | 0 | 1 | 1 | **4** |
| **Almandoz JP et al. 2020 (61)** | 0 | 0 | 0 | 1 | 2 | 1 | 1 | **5** |
| **Song L et al. 2020 (54)** | 1 | 0 | 0 | 1 | 1 | 1 | 1 | **5** |
| **Grabia et al 2020 (28)** | 0 | 0 | 0 | 2 | 2 | 1 | 1 | **5** |
| **Błaszczyk-Bębenek E et al. 2020 (26)** | 1 | 0 | 0 | 2 | 0 | 1 | 1 | **5** |
| **Hanke et al 2020 (48)** | 0 | 0 | 0 | 2 | 0 | 2 | 1 | **5** |
| **Wilson et al 2020 (55)** | 0 | 0 | 0 | 1 | 2 | 1 | 1 | **5** |
| **Nienhuis and Lesser, 2020 (56)** | 1 | 0 | 0 | 1 | 1 | 1 | 1 | **5** |
| **Gualano MR et al. 2020 (62)** | 1 | 0 | 0 | 1 | 2 | 1 | 1 | **6** |
| **Wanberg et al 2020 (57)** | 1 | 0 | 0 | 1 | 2 | 1 | 1 | **6** |
| **Zenic et al. 2020 (43)** | 0 | 0 | 0 | 2 | 2 | 1 | 1 | **6** |
| **Guo et al 2020 (53)** | 0 | 0 | 0 | 2 | 2 | 1 | 1 | **6** |
| **Elran-Barak R, & Mozeikov M. 2020 (71)** | 1 | 0 | 0 | 1 | 2 | 1 | 1 | **6** |
| **Fernandez-Rio et al 2020 (21)** | 1 | 1 | 1 | 2 | 2 | 1 | 0 | **6** |
| **Lopez-Moreno et al 2020 (33)** | 1 | 0 | 0 | 2 | 1 | 1 | 1 | **6** |
| **Gallè F et al. 2020 (25)** | 0 | 1 | 1 | 2 | 0 | 1 | 1 | **6** |
| **Flentje A et al. 2020 (92)** | 0 | 0 | 1 | 2 | 1 | 1 | 1 | **6** |
| **Sassone et al.,2020 (43)** | 0 | 0 | 0 | 2 | 1 | 2 | 1 | **6** |
| **Tornaghi et al 2020 (47)** | 0 | 0 | 0 | 2 | 2 | 1 | 1 | **6** |
| **Hemphill NM et al. 2020 (36)** | 0 | 0 | 0 | 2 | 1 | 2 | 1 | **6** |
| **Huckins JF et al. 2020 (37)** | 0 | 0 | 1 | 1 | 1 | 2 | 1 | **6** |
| **Bourdas et al (2020b) (38)** | 1 | 0 | 0 | 1 | 2 | 1 | 1 | **6** |
| **Muriel et al (2020) (40)** | 1 | 0 | 0 | 2 | 1 | 1 | 1 | **6** |
| **Savage et al (2020) (41)** | 1 | 0 | 1 | 2 | 0 | 1 | 1 | **6** |
| **Evanoff et al 2020 (52)** | 1 | 0 | 0 | 1 | 2 | 1 | 1 | **6** |
| **McDowell et al 2020 (59)** | 1 | 0 | 0 | 1 | 2 | 1 | 1 | **6** |
| **Garcia-Alvarez et al 2020 (60)** | 1 | 0 | 0 | 1 | 2 | 1 | 1 | **6** |
| **Robinson et al 2020 (81)** | 0 | 0 | 0 | 2 | 2 | 1 | 1 | **6** |
| **Do et al 2020 (82)** | 1 | 1 | 0 | 2 | 0 | 1 | 1 | **6** |
| **Huber et al 2020 (63)** | 0 | 0 | 0 | 2 | 2 | 1 | 1 | **6** |
| **Sidor A, & Rzymski P. 2020 (23)** | 1 | 0 | 0 | 1 | 2 | 1 | 1 | **6** |
| **Górnicka M et al. 2020 (68)** | 1 | 0 | 0 | 1 | 2 | 1 | 1 | **6** |
| **Rodríguez-Pérez C et al. 2020 (77)** | 1 | 0 | 0 | 2 | 2 | 1 | 0 | **6** |
| **Yan AF et al. 2020 (78)** | 1 | 0 | 0 | 2 | 1 | 1 | 1 | **6** |
| **Visser et al 2020 (64)** | 0 | 0 | 0 | 2 | 2 | 1 | 1 | **6** |
| **Pietrobelli A et al. 2020 (67)** | 0 | 0 | 0 | 2 | 2 | 1 | 1 | **6** |
| **Ammar et al 2020 (83)** | 1 | 1 | 1 | 1 | 0 | 1 | 1 | **6** |
| **Munk AJL et al. 2020 (89)** | 1 | 0 | 0 | 2 | 2 | 1 | 1 | **7** |
| **Vetrovsky et al. 2020 (42)** | 0 | 0 | 0 | 2 | 2 | 2 | 1 | **7** |
| **Ismail et al 2020 (22)** | 1 | 1 | 1 | 2 | 0 | 1 | 1 | **7** |
| **Pišot et al 2020 (27)** | 1 | 1 | 1 | 2 | 0 | 1 | 1 | **7** |
| **Yang et al 2020 (29)** | 1 | 0 | 1 | 2 | 1 | 1 | 1 | **7** |
| **Jia et al 2020 (32)** | 1 | 0 | 1 | 2 | 1 | 1 | 1 | **7** |
| **Martinez-de-Quel et al 2020 (31)** | 1 | 0 | 0 | 2 | 2 | 1 | 1 | **7** |
| **Mason et al 2020 (34)** | 1 | 1 | 0 | 2 | 2 | 1 | 1 | **7** |
| **Kannampallil et al 2020 (94)** | 1 | 0 | 0 | 2 | 2 | 1 | 1 | **7** |
| **Liu et al 2020 (96)** | 1 | 0 | 0 | 2 | 2 | 1 | 1 | **7** |
| **Pieh et al. 2020 (88)** | 1 | 0 | 0 | 2 | 2 | 1 | 1 | **7** |
| **Chen et al 2020 (85)** | 1 | 0 | 0 | 2 | 2 | 1 | 1 | **7** |
| **Xiang et al 2020 (95)** | 1 | 0 | 0 | 2 | 2 | 1 | 1 | **7** |
| **Wanberg et al 2020 (57)** | 1 | 0 | 0 | 2 | 2 | 1 | 1 | **7** |
| **Cai et al 2020 (98)** | 1 | 0 | 0 | 2 | 2 | 1 | 1 | **7** |
| **Coughenour et al 2020 (86)** | 1 | 0 | 0 | 2 | 2 | 1 | 1 | **7** |
| **Zheng et al 2020 (45)** | 1 | 0 | 0 | 2 | 2 | 1 | 1 | **7** |
| **Schmidt et al 2020 (46)** | 1 | 0 | 0 | 2 | 2 | 1 | 1 | **7** |
| **Munasinghe et al (2020) (39)** | 1 | 0 | 1 | 2 | 1 | 1 | 1 | **7** |
| **Donnelly et al 2020 (58)** | 1 | 1 | 0 | 1 | 2 | 1 | 1 | **7** |
| **Carroll et al 2020 (84)** | 1 | 0 | 0 | 2 | 2 | 1 | 1 | **7** |
| **Buckland et al 2020 (65)** | 1 | 0 | 0 | 2 | 2 | 1 | 1 | **7** |
| **Di Renzo L et al. 2020 (66)** | 1 | 0 | 0 | 2 | 2 | 1 | 1 | **7** |
| **Almandoz JP et al. 2020 (61)** | 1 | 1 | 0 | 1 | 2 | 1 | 1 | **7** |
| **Ruiz-Roso MB et al. 2020(69)** | 1 | 0 | 0 | 2 | 2 | 1 | 1 | **7** |
| **Wang X et al. 2020 (70)** | 1 | 0 | 0 | 2 | 2 | 1 | 1 | **7** |
| **Knell G et al. 2020 (73)** | 1 | 0 | 1 | 1 | 2 | 1 | 1 | **7** |
| **Malta DC et al. 2020 (76)** | 1 | 0 | 1 | 2 | 2 | 1 | 0 | **7** |
| **Ruiz-Rozo et al, 2020 (79)** | 1 | 0 | 0 | 2 | 2 | 1 | 1 | **7** |
| **Yang Y, & Koenigstorfer J. 2020 (49)** | 1 | 1 | 0 | 2 | 2 | 1 | 1 | **8** |
| **De Luis Roman et al 2020 (30)** | 1 | 1 | 1 | 2 | 2 | 1 | 1 | **8** |
| **Pellegrini M et al. 2020 (24)** | 1 | 1 | 1 | 2 | 2 | 1 | 1 | **8** |
| **Ettman CK et al 2020 (93)** | 1 | 1 | 0 | 2 | 2 | 1 | 1 | **8** |
| **Quittkat et al 2020 (97)** | 1 | 1 | 1 | 2 | 1 | 1 | 1 | **8** |
| **Schmitz et al. 2020 (90)** | 1 | 1 | 0 | 2 | 2 | 1 | 1 | **8** |
| **Li et al 2020 (100)** | 1 | 1 | 0 | 2 | 2 | 1 | 1 | **8** |
| **Elmer T et al. 2020 (87)** | 1 | 0 | 1 | 2 | 2 | 1 | 1 | **8** |
| **Sánchez-Sánchez E et al. 2020 (72)** | 1 | 1 | 0 | 2 | 2 | 1 | 1 | **8** |
| **Gallo LA et al. 2020(50)** | 1 | 0 | 0 | 2 | 2 | 2 | 1 | **8** |
| **Husain W, & Ashkanani F. 2020 (74)** | 1 | 1 | 0 | 2 | 2 | 1 | 1 | **8** |
| **Alhusseini and Alqahtani, 2020 (80)** | 1 | 1 | 0 | 2 | 2 | 1 | 1 | **8** |
| **Steele EM et al. 2020 (75)** | 1 | 1 | 0 | 2 | 2 | 1 | 1 | **8** |
| **Thombs et al 2020 (99)** | 1 | 1 | 1 | 2 | 2 | 1 | 1 | **9** |
| **Wang et al.,2020 (35)** | 1 | 1 | 0 | 2 | 2 | 2 | 1 | **9** |

| **Criteria** | **Acceptable (star awarded)** | **Unacceptable (star not awarded)** |
| --- | --- | --- |
| Representativeness of exposed cohort | Population-based with random or non-random sampling | Specific cohort/selected group |
| Sample size | Justified and satisfactory size | Not justified (not sufficiently powered) or unsatisfactory size |
| Non-respondents | Response rate satisfactory  Comparison between respondent and non-respondent characteristics is established | Different setting from exposed cohort  No mention of comparison between respondent and non-respondent characteristics |
| Ascertainment of exposure | Secure records, direct or indirectly measured | Self-reported information |
| Comparability | Controls for potential confounding factors (such as age, sex, exposure to coronavirus) or takes into account as limiting factor of study | Does not control for potential confounding factors or no mention of these |
| Outcome of interest | Two stars – independent blind assessment or secure records  One star – Self-reported data | No description of how outcome measure obtained |
| Statistical test | Statistical test of choice is described and appropriate, confidence intervals and probability level (p value) are stated | No description of statistical test, no inclusion of confidence intervals or probability level |
